# Proof-of-concept of bayesian latent class modelling usefulness for assessing diagnostic tests in absence of diagnostic standards in mental health

**DOI:** 10.1038/s41598-025-17332-3

**Published:** 2025-10-02

**Authors:** Yara Shoman, Sonja Hartnack, Céline Leclercq, Isabelle Hansez, Irina Guseva Canu

**Affiliations:** 1 Center of primary care and public health (Unisanté), University of Lausanne, Lausanne, 1066 Switzerland; 2 Section of Epidemiology, Vetsuisse Faculty, University of Zurich, Zurich, 8057 Switzerland; 3 Human Resources Development Unit, Faculty of Psychology, Language Therapy and Education, University of Liège, Liège, 4000 Belgium; 4 Centre for Veterinary Systems Transformation and Sustainability Clinical Department for Farm Animals and Food System Science, University of Veterinary Medicine Vienna, Vienna, 1210 Austria

**Keywords:** Bayesian latent class modelling, Burnout, Prevalence, Sensitivity, Specificity, Psychology, Epidemiology, Medical research, Outcomes research

## Abstract

This study aimed at demonstrating the feasibility, utility and relevance of the Bayesian Latent Class Modelling (BLCM), not assuming a gold standard, when assessing the diagnostic accuracy of the first hetero-assessment test for early detection of occupational burnout (EDTB) by healthcare professionals and the OLdenburg Burnout Inventory (OLBI). We used available data from OLBI and EDTB completed for 100 Belgian and 42 Swiss patients before and after medical consultations. We applied the Hui-Walter framework for two tests and two populations and ran models with minimally informative priors, with and without conditional dependency between diagnostic sensitivities and specificities. We further performed sensitivity analysis by replacing one of the minimally informative priors with the distribution beta^1,2^ at each time for all priors. We also performed the sensitivity analysis using literature-based informative priors for OLBI. Using the BLCM without conditional dependency, the diagnostic sensitivity and specificity of the EDTB were 0.91 (0.77-1.00) and 0.82 (0.59-1.00), respectively. The sensitivity analysis did not yield any significant changes in these results. The EDTB’s sensitivity and specificity obtained by a BLCM approach are better compared to the previous studies when EDTB was evaluated against OLBI, considered as a gold standard. These findings show the utility and relevance of BLCM in the absence of a gold standard.

**Supplementary Information:**

The online version contains supplementary material available at 10.1038/s41598-025-17332-3.

## Introduction

 Diagnosis and assessment of health outcomes hold valuable impact on healthcare, medical research, and policy^1^. A perfect diagnostic test has the possibility to entirely differentiate people with and without the outcome of interest (i.e., the disease), without any misclassifications^2^. A perfect diagnostic test, a gold standard, does not exist in a real life setting and thus diagnostic tests could only partially differentiate people with and without disease^2^. The estimation of diagnostic accuracy of health outcome tests can be performed by estimating the diagnostic sensitivity, specificity, and positive and negative predictive values^3^. Estimating the diagnostic sensitivity and specificity requests a diagnostic standard, which serves as a reference^4^. In the case of imperfect tests, i.e. diagnostic sensitivity and/or specificity being lower than 100%, misclassifications will occur. However, for many outcomes, such diagnostic standards are lacking. This is particularly true in mental health and subjective outcome measures^5^. The lack of standard diagnostic or screening criteria makes it challenging to assess the validity of newly developed or existing tests^6^, leading typically to underestimations of their diagnostic accuracy as all discrepancies in test results between the new and the standard test will always be considered as misclassifications of the new test^7^. To address this issue, Bayesian Latent Class Modelling (BLCM) was proposed to assess the performance of diagnostic tests in the absence of a perfect gold standard^8^.

Following a BLCM approach, we can observe the combination of observed diagnostic test outcomes and accuracy and prior knowledge of the outcome prevalence to obtain a posterior knowledge of the accuracy of the diagnostic tests in the populations of interest^9^. In summary, prior information about the diagnostic sensitivity and specificity is linked with observed data (e.g. diagnostic test outcomes) to acquire a posterior distribution of the variable^10^. Hui and Walter^11^ presented the first Latent Class Analysis (LCA) model to estimate diagnostic test accuracy using two imperfect tests applied to two populations with different outcome prevalence. An important method that increased the implementation of Bayesian statistics, in the case of lack of a gold standard and use of imperfect tests, is the Gibbs sampling^14^. Gibbs sampling is a Markov Chain Monte Carlo algorithm which uses simulations to manage a complex integration process essential for a full Bayesian analysis. The first user-friendly Bayesian statistical software package using Gibbs sampling was “WinBUGS”. WinBUGS permitted the user to indicate the likelihood for the data and the distributions of the prior for each parameter in the model allowing to derive the posteriors. With the WinBUGS package, the Hui − Walter model could be estimated in a Bayesian framework with a user-friendly interface. Later, other statistical software packages based on this methodology (e.g., JAGS, OpenBUGS) were introduced^8^. A key assumption of the original Hui − Walter model is (I) that sensitivity and specificity are the same in all populations, and (II) that the diagnostic tests under assessment are independent and conditional on the true outcome status of a person^11^. However, these assumptions are rather difficult to validate in most cases. Soon after the Hui-Walter model was first described, the effect of conditional dependence between diagnostic tests on the evaluation of diagnostic accuracies, i.e. under- or overestimated was stated^12^. BLCM models that model conditional dependence between tests using covariance terms were later developed to soften Hui-Walter’s assumption of conditional independence^13,14^. These more complex BLCM models allow for more precise estimation of diagnostic test accuracies^8^.

In this paper we will use the example of two diagnostic tests to assess burnout as an illustration of the usefulness of BLCM application in the absence of a gold standard in mental health research. Burnout is an interesting example because despite extensive research, no consensus still exists regarding its measurement^15^, similar to other psychiatric and psychological phenomena. Currently, burnout is mainly evaluated through Patient-Reported Outcome Measures (PROMs). PROMs measure a patient’s health status or health-related quality of life at a single point in time, and are collected through short, self-completed questionnaires. Oldenburg Burnout Inventory (OLBI) is one of the widely used available PROMs to assess burnout^16^. Yet, to improve and facilitate the burnout measurement by health professionals, the Early Detection Tool of Burnout (EDTB) was recently developed as the first published hetero-assessment test^17^. Hetero-assessment instruments are filled out by one person (i.e., physician or clinical psychologist) about another (i.e., patient) and encompass evaluations of the individual’s work capacity, attitude, performance, and other characteristics^18^.

We have previously assessed the diagnostic accuracy of EDTB in Belgian and Swiss study samples, using OLBI as a comparator assumed to be a gold standard^19,20^. The Belgian and Swiss studies were independent, each having its own study sample, but used the same tests. However, these studies suffer from an important limitation due to the lack of a gold standard test for measuring burnout, which may introduce bias when comparing EDTB’s performance to an imperfect comparator (OLBI). In the present study, we used a completely different methodological approach (LCM) and analytical paradigm (Bayesian) that we applied to study sample comprising both Belgian and Swiss samples merged. This study aims to demonstrate the advantages of using BLCM by comparing the diagnostic accuracy of two burnout assessment tests without considering any of them as a reference test.

## Results

The results of BLCM showed no difference between models with and without conditional dependencies (Supplementary Tables S1 & S2). The 95% credibility intervals of covariance terms to model conditional dependencies between diagnostic sensitivities and specificities included 0. Using the BLCM without conditional dependencies, the diagnostic sensitivity and specificity of EDTB were 0.91 and 0.82, respectively (Table 1). The diagnostic sensitivity and specificity of OLBI were 0.73 and 0.73, respectively (Table 1). The estimated prevalence of burnout was 52% in the Belgian study population and 82% in the Swiss study population. The effective sample size (SSeff > 1000) and Gelman-Rubin statistic (psrf < 1.05) validated the model fit (Table 1). By plotting the results, we observed that the two chains converged well for the diagnostic sensitivity and specificity of EDTB and OLBI (Supplementary Figures S1 & S2). We also observed that the autocorrelations decreased after a small number of iterations (Supplementary Figures S1 & S2). The estimated Positive Predictive Value (PPV) in the Belgian population was 84.5% and the estimated Negative Predictive Value (NPV) was 89%. The estimated PPV in the Swiss population was 95.8% and the estimated NPV was 66%.

**Table 1:** Results of the bayesian latent class modelling using minimally informative priors and without conditional dependency.

	Estimate	Lower 95% credibility interval	Upper 95% credibility interval	The effective sample size (SSef)	Gelman-Rubin statistic (psrf)
Diagnostic sensitivity of the EDTB	0.905	0.772	1.000	26,331	1
Diagnostic specificity of the EDTB	0.822	0.589	1.000	22,704	1
Diagnostic sensitivity of OLBI	0.730	0.593	0.892	24,468	1
Diagnostic specificity of OLBI	0.726	0.544	0.936	26,124	1
Prevalence (Belgian study population)	0.518	0.293	0.713	21,932	1
Prevalence (Swiss study population)	0.817	0.584	0.999	23,211	1

* EDTB: Early Detection Tool of Burnout,* OLBI: Oldenburg Burnout Inventory*.

The sensitivity analysis did not yield changes in the diagnostic sensitivity and specificity of EDTB or OLBI (Supplementary material S3-S10). In all models, the diagnostic sensitivity was never below 0.82 and the diagnostic specificity was never below 0.78. Yet, in some models (Supplementary material Table S5), the credibility intervals for the diagnostic specificity of EDTB became wider. The lowest estimated diagnostic sensitivity of OLBI was 0.69 whereas 0.67 was the lowest estimated diagnostic specificity of OLBI.

## Discussion

We contrasted the results obtained through the BLCM approach used in this study with those from the conventional approach applied in the earlier studies using the classical gold standard approach^18–20^. Thus, this study aimed to illustrate the application of BLCM in diagnostic mental health research when a gold standard is lacking. For this, we used burnout measurement as example and assessed the diagnostic performance of two burnout measures: EDTB and OLBI independently. The findings of the present study indicate that BLCM is a relevant and feasible approach that helps evaluate the diagnostic sensitivity and specificity of tests particularly in the absence of gold standards. The model performance was sufficient, and the two chains converged well, and the Gelman-Rubin statistics indicated convergence which indicate the reliability of the results despite the small sample size. Based on this study results, the diagnostic sensitivity and specificity of the EDTB are better compared to the estimates reported in previous studies that used the classical approach assuming that the reference test is a gold standard, i.e., being 100% sensitive and specific. In the Swiss study^20^, the reported diagnostic sensitivity and specificity of the EDTB were 0.88 and 0.29, respectively. In the Belgian study^19^, these estimates were 0.76, and 0.60, respectively. Regarding the diagnostic accuracy of OLBI, the estimated diagnostic sensitivity and specificity in this study was slightly higher compared to the Belgian study (0.70, 0.67)^19^, whereas the Swiss study did not report these values precluding the comparison^20^. The estimated diagnostic sensitivity and specificity of the EDTB and OLBI using BLCM encourage their use in the assessment of diagnostic accuracy of health outcome measures for which there is no diagnostic standard.

This study provided a proof of concept of the use of BLCM in the absence of a gold standard, which is often the case for mental health outcomes. For example, the quality of life outcome assessment is difficult because this outcome is a latent variable which is not directly observable and there is no gold standard for its assessment^21^. The example of burnout assessment presented in this paper is especially sound because this phenomenon has significant consequences both at the individual and organizational levels^22^. The standardization of burnout detection is imperative to support high-quality research that facilitates evidence-based interventions to protect workers and reduce this phenomenon^23^. The importance of utilizing standardized methods in assessment is widely recognized as the foundation of evidence-based practice^24^. However, the current assessment of burnout is mainly reliant on self-reported instruments^16^ and therefore there is a need for a structured interview instrument (i.e., EDTB) for the prognostic and interventional activity and research on burnout. Hence, a re-assessment of the diagnostic accuracy of EDTB is still essential with larger representative samples.

The study aimed to demonstrate the feasibility and relevance of BLCM in burnout research by utilizing data from two previous studies with small study samples. This constitutes a limitation of the study, as it may impact the precision of the results. The assumption that the diagnostic sensitivity and specificity of the two burnout assessment measures were comparable across the Swiss and Belgian populations could also be considered as a limitation. However, considering the similarities between the Swiss and Belgian populations, we may assume that the diagnostic sensitivity and specificity of EDTB and OLBI were comparable. This study serves as a proof of concept and focuses on replacing the practice of gold standard assumption when determining the accuracy of a new test. Nevertheless, performing a thorough quality and validity assessment of the tests remains crucial although it is beyond the scope of this paper.

The question of whether burnout should be considered a continuous or dichotomous variable still remains open^25^. In our data set, we used dichotomous test results, but BLCM approaches modeling continuous test results are also available^26^. Schaufeli et al., already discussed the question of continuous versus dichotomous results in 2008 “*Medical practitioners favor dichotomous diagnoses*,* especially when informing decisions on treatment or disability insurance claims. In this way the definition of burnout is shaped by practical questions– Who is to be treated? Who is to receive financial compensation?”*^27^. Schaufeli et al., described the dichotomization of burnout as an expansion from a psychological phenomenon to a medical diagnosis at least in some European countries such as The Netherlands and Sweden and using statistical or clinically validated cut-offs^27^. More recently, a study by Guseva Canu et al. showed that 14 out of 34 European and neighboring countries including Turkey, Denmark, Lithuania, to cite just a few, recognize and compensate burnout as an occupational disease^28^. Finally, the most recently developed burnout measurement test (Burnout Assessment Tool) provided clinical cut-off values to facilitate dichotomization and case identification^29^. The debate on dichotomization of an evolving condition is not unique to burnout but shared by several psychiatric and psychological phenomena in mental health research e.g., psychosis^29^.

It is important to highlight the underlying assumption of the BLCM that a positive (“+”) or negative (“−“) result holds the same interpretation for both the OLBI and the EDTB, an assumption that remains open to debate. Strictly speaking, however, this assumption is also made in the classical approach in diagnostic test evaluation, when a new test is evaluated against an assumed perfect reference test. In contrast to this, BLCM is a more realistic approach, allowing the reference test to be imperfect. Moreover, the cut-offs we used to dichotomize burnout measured using OLBI could be considered arbitrary and future research should address this issue either by validating clinical cut-offs or using continuous scales in BLCM.

This study provided a practical example of the feasibility and relevance of BLCM for diagnostic research. To our knowledge, this is the first study conducted using BLCM to assess the diagnostic sensitivity and specificity of two burnout measures. The findings of this study encourage the use of BLCM in future, methodologically sound studies and for other outcomes particularly subjectively measured ones such as: quality of life, and wellbeing.

The EDTB’s diagnostic sensitivity and specificity re-assessed using BLCM are better and less biased compared to the previous studies conducted using the classical gold standard approach. This study demonstrated the practical feasibility and scientific relevance of BLCM for diagnostic research especially for subjectively measured outcomes for which there are no standard diagnostic criteria, such as burnout.

## Methods

### Study populations

The present study utilized data from two previous studies^19,20^. The population 1 consisted of 42 Swiss patients (mean age 46 years, 74% women), who completed the OLBI before their “Work and Suffering” consultation at Unisanté between 2010 and 2013 and for whom the EDTB was completed retrospectively by the occupational physicians in charge of the consultation, based on their clinical reports. The population 2 comprised 123 Belgian patients (mean age 44 years, 50% women), who consulted an occupational physician (*n* = 100) or a general practitioner (*n* = 23) in 2018. The physicians had to complete the EDTB online, either during or right after the consultation. In addition to this clinical judgment tool, physicians asked each patient to complete a paper version of the OLBI after the consultation. The results of the Belgian study showed that occupational physicians had better estimates of diagnostic sensitivities and specificities compared to general practitioners or both types of physicians. As a result, we used data from 100 Belgian and 42 Swiss patients who had a consultation with an occupational physician. Both the Belgian and Swiss studies utilized convenience sampling as the recruitment method^19,20^.

### Occupational burnout tests

The EDTB: In 2020, the Belgian Federal Public Service Employment, Labour and Social Dialogue (BFPSELSD) introduced the first tool for early detection of occupational burnout (EDTB) to hetero-assess burnout by health professionals^17^. Two recent studies^19,20^ have examined the diagnostic accuracy of the EDTB by comparing it to a valid self-reported burnout test, the Oldenburg Burnout Inventory (OLBI)^30^. The EDTB is a concise three-page document that encompasses various topics^17^. These include general complaints such as sleep disturbances, stress, workload, and workplace conflicts; symptoms of burnout across physical, cognitive, emotional, and behavioral domains; work-related factors like the origins of complaints, potential risk elements, job demands, and available job resources; sociodemographic details; data on absenteeism; specific diagnoses like burnout, anxiety, and depression; and any additional remarks. Health professionals have the option to select one or multiple items under each category based on the worker’s feedback. Ultimately, the health professional’s conclusion, specifically whether burnout is present or absent, serves as the primary piece of information^17^.

The EDTB was validated in French and Dutch languages^19^.

OLBI consists of two dimensions^30^: exhaustion (8 items) and disengagement (8 items). The items of OLBI are scored on a Likert scale where responses range from 1 to 4, typically with “Strongly agree” at one end and “Strongly disagree” at the other end. Example of items are: “I always find new and interesting aspects in my work” and “During my work, I often feel emotionally drained”. The sum of the responses is calculated, given that each item has four possible answers (1, 2, 3 or 4), we obtain a score ranging from 16 to 64. To obtain a dichotomized status regarding burnout, the Swiss and Belgian studies used a cut-off of 44, which means that scores higher than 44 represent high-severity burnout. This cut-off is based on the findings from the previous studies, because OLBI does not classify burnout in a dichotomous outcome but instead provides a global score ranging from 16 to 64. In our previous analysis^20^, we evaluated two cut-offs values of OLBI and decided to use the cut-off of 44 because it was more reliable according to the Power et al. criteria^31^. The validity and reliability of OLBI was reported to be sufficient in a recent systematic review^16^. There are Dutch and French validated versions of OLBI which were used in this study^32^.

 There was a fair overall agreement between OLBI and EDTB, with a significant but fair to moderate kappa (κ = 0.36, 95%CI = 0.20, 0.54)^19^.

### Analysis

We applied BLCM using the Hui-Walter framework^11^ with Markov Chain Monte Carlo (MCMC) simulation to two tests and two populations. We used minimally informative priors in models, with or without conditional dependencies between diagnostic sensitivities of OLBI and EDTB or specificities of OLBI and EDTB. The model code (Supplementary material) was obtained with the function “auto hui-walter” of the runjags package^33^ using the freely available software R (R Core Team, 2024 Version-4.4.1, https://www.r-project.org/). We set the burn-in to 5000 and the sample to 10,000 iterations for two independent chains. To assess convergence, we plotted the trace plots and considered the potential scale reduction factor (< 1.05). Model selection, i.e., in- or exclusion of conditional dependencies was based on the 95% credibility intervals (including 0 or not). The prevalence we measure in this study is the prevalence in our study population and not in the general population. Indeed, out target population are workers exposed to job stress and therefore at risk of burnout. Our source population is the workers who came to the consultation either at Unisanté center of occupational medicine in Switzerland or during general practitioners and occupational physician’s consultations for work-related health problems in Belgium and who completed the OLBI test of burnout. We computed Positive Predictive Value (PPV) and Negative Predictive Value (NPV) for Belgian and Swiss populations. PPV was computed for a test (i) in a population (k) using the formula: P_k_​Se_ik_​​/(P_k​_Se_ik_​+(1 − P_k_​)(1 − Sp_ik_​)). NPV was calculated using the formula: ​(1 − P_k_​)Sp_ik_/(P_k_​(1 − Se_ik_​)+(1 − P_k_​)Sp_ik_).

We further performed sensitivity analysis by replacing one of the minimally informative priors with the distribution^1,2^ at each time for the six priors. These six priors were: diagnostic sensitivity and specificity of EDTB, diagnostic sensitivity and specificity of OLBI and prevalence of burnout in each study population (Swiss and Belgian samples). Moreover, we performed the analysis using informative priors for OLBI based on the data from the literature^34,35^.

We reported the results of this study following the Standards for the Reporting of Diagnostic accuracy studies that use Bayesian Latent Class Models (STARD-BLCM) guidelines^36,37^. All codes and analyses are available online from: DOI:10.16909/DATASET/35.

## Supplementary Information

Below is the link to the electronic supplementary material.

Supplementary Material 1

Supplementary Material 2

## Data Availability

All codes, supplementary figures and tables are available from the link: doi:10.16909-DATASET-35.

## References

[1] Committee on Diagnostic Error in Health Care; Board on Health Care Services; Institute of Medicine; The National Academies of Sciences E, and Medicine. in *The Diagnostic Process*. (eds Balogh, E., Miller, B. & Ball, J.) (National Academies, 2019).

[2] Šimundić, A. M. Measures of diagnostic accuracy: basic definitions. *Ejifcc* **19** (4), 203–211 (2009). 27683318 PMC4975285

[3] Santini, A., Man, A. & Voidăzan, S. Accuracy of diagnostic tests. *J. Crit. Care Med. (Targu Mures)*. **7** (3), 241–248 (2021). 34722928 10.2478/jccm-2021-0022 PMC8519382

[4] Dohoo, I. R., Martin, W. & Stryhn, H. E. Veterinary epidemiologic research (2003).

[5] Breedvelt, J. J. F. et al. A systematic review of mental health measurement scales for evaluating the effects of mental health prevention interventions. *Eur. J. Pub. Health*. **30** (3), 510–516 (2020). 32236548 10.1093/eurpub/ckz233

[6] Schofield, M. R. et al. On the robustness of latent class models for diagnostic testing with no gold standard. *Stat. Med.* **40** (22), 4751–4763 (2021). 33990992 10.1002/sim.8999 PMC8440412

[7] Umemneku Chikere, C. M., Wilson, K. J., Allen, A. J. & Vale, L. Comparative diagnostic accuracy studies with an imperfect reference standard – a comparison of correction methods. *BMC Med. Res. Methodol.* **21** (1), 67 (2021). 33845775 10.1186/s12874-021-01255-4 PMC8040223

[8] Cheung, A. et al. Bayesian latent class analysis when the reference test is imperfect. *Rev. Sci. Tech. Oie*. **40** (1), 271–286 (2021). 10.20506/rst.40.1.3224 34140724

[9] Joseph, L., Gyorkos, T. W. & Coupal, L. Bayesian Estimation of disease prevalence and the parameters of diagnostic tests in the absence of a gold standard. *Am. J. Epidemiol.* **141** (3), 263–272 (1995). 7840100 10.1093/oxfordjournals.aje.a117428

[10] Branscum, A. J., Gardner, I. A. & Johnson, W. O. Estimation of diagnostic-test sensitivity and specificity through bayesian modeling. *Prev. Vet. Med.* **68** (2–4), 145–163 (2005). 15820113 10.1016/j.prevetmed.2004.12.005

[11] Hui, S. L. & Walter, S. D. Estimating the error rates of diagnostic tests. *Biometrics* **36** (1), 167–171 (1980). 7370371

[12] Vacek, P. M. The effect of conditional dependence on the evaluation of diagnostic tests. *Biometrics* **41** (4), 959–968 (1985). 3830260

[13] Dendukuri, N. & Joseph, L. Bayesian approaches to modeling the conditional dependence between multiple diagnostic tests. *Biometrics* **57** (1), 158–167 (2001). 11252592 10.1111/j.0006-341x.2001.00158.x

[14] Georgiadis, M. P., Johnson, W. O. & G, I. A. Correlation-adjusted Estimation of sensitivity and specificity of two diagnostic tests. *J. Roy Stat. Soc. C (Appl Stat)*. **52** (1), 63–76 (2003).

[15] Nadon, L., De Beer, L. T. & Morin, A. J. S. Should burnout be conceptualized as a mental disorder?? behavioral sciences (Basel. *Switzerland)* ;**12**(3), 11 (2022). 10.3390/bs12030082 PMC8945132 35323401

[16] Shoman, Y., Marca, S. C., Bianchi, R., Godderis, L. & van der Molen, H. F. Guseva Canu I. Psychometric properties of burnout measures: a systematic review. *Epidemiol. Psychiatric Sci.* **30**, e8 (2021). 10.1017/S2045796020001134 PMC8057391 33436137

[17] Fédéral, S. P. Détection Précoce du Burnout: Outil Pour le Médecin Généraliste Burnout (Early Detection of Burnout: BPSEWSTool for the General Physician) 2020 [Available from: Available online: https://www.souffrance-et-travail.com/magazine/burn-out/outil-de-detection-precoce-du-burn-out/

[18] Casanova, M. La evaluación educativa: escuela básica. México, DC: Muralla-SEP; (1998). Available from: http://ed.dgespe.sep.gob.mx/materiales/primaria/competencias.didacticas/la.evaluacion.educativa.educacion.basica.pdf

[19] Leclercq, C., Braeckman, L., Firket, P., Babic, A. & Hansez, I. Interest of a joint use of two diagnostic tools of burnout: comparison between the Oldenburg burnout inventory and the early detection tool of burnout completed by physicians. *Int. J. Environ. Res. Public Health*. **18**, 19 (2021). 10.3390/ijerph181910544 PMC8507986 34639844

[20] Nguyen Huynh, A. et al. Diagnostic performances of an occupational burnout detection method designed for healthcare professionals. *Int. J. Environ. Res. Public Health* ;**18**(23), 2 (2021). 10.3390/ijerph182312300 PMC8657176 34886022

[21] Blome, C. & Augustin, M. Measuring change in quality of life: bias in prospective and retrospective evaluation. *Value Health*. **18** (1), 110–115 (2015). 25595241 10.1016/j.jval.2014.10.007

[22] Edú-Valsania, S., Laguía, A., Moriano, J. A. & Burnout A review of theory and measurement. *Int. J. Environ. Res. Public Health* ;**19**(3), 10 (2022). 10.3390/ijerph19031780 PMC8834764 35162802

[23] Chirico, F., Nucera, G. & Leiter, M. Measuring burnout syndrome requires reliable and standardized measures. *Hong Kong J. Emerg. Med.* **29**, 102490792210969 (2022).

[24] Lyon, A. R. et al. Determinants and functions of standardized assessment use among school mental health clinicians: A mixed methods evaluation. *Adm. Policy Ment. Health*. **43** (1), 122–134 (2016). 25875325 10.1007/s10488-015-0626-0 PMC4500742

[25] Doulougeri, K., Georganta, K. & Montgomery, A. Diagnosing burnout among healthcare professionals: can we find consensus? *Cogent Med.* **3** (1), 1 (2016).

[26] Wang, Y. et al. A novel bayesian latent class model (BLCM) evaluates multiple continuous and binary tests: A case study for Brucella abortus in dairy cattle. *Prev. Vet. Med.* **224**, 106115 (2024). 38219433 10.1016/j.prevetmed.2024.106115

[27] Schaufeli, W. B., Leiter, M. P. & Maslach, C. Burnout: 35 years of research and practice. *Career Dev. Int.* **14** (3), 204–220 (2009).

[28] Guseva Canu, I. et al. Burnout syndrome in europe: towards a harmonized approach in occupational health practice and research. *Ind. Health*. **57** (6), 745–752 (2019). 30814391 10.2486/indhealth.2018-0159 PMC6885602

[29] SchaufeliWB, De WitteH, HakanenJJ, Kaltiainen, J. & Kok, R. How to assess severe burnout? *Scand. J. Work. Environ. Health.* **4**, 293–302 (2023). Cutoff points for the Burnout Assessment Tool (BAT) based on three European samples. 10.5271/sjweh.4093 PMC10713992 37042446

[30] Demerouti, E., Bakker, A. B., Nachreiner, F. & Schaufeli, W. B. The job demands-resources model of burnout. *J. Appl. Psychol.* **86** (3), 499 (2001). 11419809

[31] Power, M., Fell, G. & Wright, M. Principles for high-quality, high-value testing. *Evid. Based Med.* **18** (1), 5 (2013). 22740357 10.1136/eb-2012-100645 PMC3585491

[32] Demerouti, E., Demerouti, E., Bakker, A. B., Vardakou, I. & Kantas, A. The convergent validity of two burnout instruments. *Eur. J. Psychol. Assess.* **19** (1), 12–23 (2003).

[33] Denwood, M. J. Runjags: an R package providing interface utilities, model templates, parallel computing methods and additional distributions for MCMC models in JAGS. *J. Stat. Softw.* **71** (9), 1–25 (2016).

[34] Campos, J., Jordani, P., Zucoloto, M., Bonafé, F. & Maroco, J. Burnout in dental students: effectiveness of different methods. *Revista De Odontologia Da UNESP*. **42**, 324–329 (2013).

[35] Chevrier, N. (ed) Adaptation québécoise de l’Oldenberg burnout inventory (OLBI)2009.

[36] Bossuyt, P. M. et al. STARD 2015: an updated list of essential items for reporting diagnostic accuracy studies. *BMJ (Clinical Res. ed)*. **351**, h5527 (2015). 10.1136/bmj.h5527 PMC4623764 26511519

[37] Kostoulas, P. et al. STARD-BLCM: standards for the reporting of diagnostic accuracy studies that use bayesian latent class models. *Prev. Vet. Med.* **138**, 37–47 (2017). 28237234 10.1016/j.prevetmed.2017.01.006

