# Supplementary material for: Proof-of-concept of bayesian latent class modelling usefulness for assessing diagnostic tests in absence of diagnostic standards in mental health
Source: Sci Rep. 2025 Oct 2;15:34398. doi: 10.1038/s41598-025-17332-3 (PMC12491510; doi:10.1038/s41598-025-17332-3)
Supplement: Supplementary file 2 — Supplementary Material 2 [file 41598_2025_17332_MOESM2_ESM.pdf]

Table S1 model with conditional dependency between sensitivities and with the use of minimally informative priors

	**Median**	**Lower CI**	**Upper CI**	**SSef**	**psrf**
Sensitivity of OLBI	0.695	0.532	0.871	17456	1
Sensitivity of the BHAI	0.880	0.696	1.000	12348	1
Specificity of OLBI	0.672	0.450	0.876	25602	1
Specificity of the BHAI	0.786	0.495	1.000	17571	1
Prevalence (Belgium)	0.520	0.268	0.757	19188	1
Prevalence (Switzerland)	0.823	0.535	1.000	20385	1
Covariance sensitivity	0.028	-0.021	0.120	13262	1
Covariance specificity	0	0	0	NA	NA

Table S2 model with conditional dependency between specificities and with the use of minimally informative priors

	**Median**	**Lower CI**	**Upper CI**	**SSef**	**psrf**
Sensitivity of OLBI	0.665	0.516	0.823	24841	1
Sensitivity of the BHAI	0.878	0.725	1.000	26623	1
Specificity of OLBI	0.560	0.398	0.838	10301	1
Specificity of the BHAI	0.630	0.406	0.969	9217	1
Prevalence (Belgium)	0.373	0.001	0.639	10939	1
Prevalence (Switzerland)	0.798	0.507	1.000	21290	1
Covariance sensitivity	0	0	0	NA	NA

Covariance specificity	0.097	-0.011	0.174	12193	1

Table S3 model without conditional dependency and with minimally informative priors but the prior for Prevalence in Belgium (2,1)

	**Median**	**Lower CI**	**Upper CI**	**SSef**	**psrf**
Sensitivity of OLBI	0.727	0.592	0.880	25155	1
Sensitivity of the BHAI	0.897	0.765	1.000	25430	1
Specificity of OLBI	0.737	0.558	0.947	26885	1
Specificity of the BHAI	0.841	0.610	1.000	23428	1
Prevalence (Belgium)	0.538	0.323	0.722	22743	1
Prevalence (Switzerland)	0.826	0.606	0.999	23965	1
Covariance sensitivity	0	0	0	NA	NA
Covariance specificity	0	0	0	NA	NA

Table S4 model without conditional dependency and with minimally informative priors but the prior for Prevalence in Switzerland (2,1)

	**Median**	**Lower CI**	**Upper CI**	**SSef**	**psrf**
Sensitivity of OLBI	0.727	0.593	0.878	25262	1
Sensitivity of the BHAI	0.900	0.768	1.000	25817	1
Specificity of OLBI	0.730	0.553	0.944	25933	1
Specificity of the BHAI	0.832	0.604	1.000	23529	1
Prevalence (Belgium)	0.525	0.316	0.722	22085	1
Prevalence (Switzerland)	0.829	0.616	1.000	23471	1
Covariance sensitivity	0	0	0	NA	NA

Covariance specificity	0	0	0	NA	NA

Table S5 model without conditional dependency and with minimally informative priors but the prior for Sensitivity of OLBI (2,1)

	**Median**	**Lower CI**	**Upper CI**	**SSef**	**psrf**
Sensitivity of OLBI	0.738	0.598	0.904	23253	1
Sensitivity of the BHAI	0.902	0.769	1.000	25191	1
Specificity of OLBI	0.735	0.560	0.953	26590	1
Specificity of the BHAI	0.816	0.582	1.000	21530	1
Prevalence (Belgium)	0.517	0.297	0.716	20484	1
Prevalence (Switzerland)	0.815	0.569	0.997	21720	1
Covariance sensitivity	0	0	0	NA	NA
Covariance specificity	0	0	0	NA	NA

Table S6 model without conditional dependency and with minimally informative priors but the prior for Specificity of OLBI (2,1)

	**Median**	**Lower CI**	**Upper CI**	**SSef**	**psrf**
Sensitivity of OLBI	0.731	0.596	0.898	24691	1
Sensitivity of the BHAI	0.899	0.765	1.000	25223	1
Specificity of OLBI	0.740	0.562	0.952	26289	1
Specificity of the BHAI	0.825	0.596	1.000	22444	1
Prevalence (Belgium)	0.524	0.316	0.726	21649	1
Prevalence (Switzerland)	0.820	0.591	0.999	22924	1
Covariance sensitivity	0	0	0	NA	NA
Covariance specificity	0	0	0	NA	NA

Table S7 model without conditional dependency and with minimally informative priors but the prior for Sensitivity of the BHAI (2,1)

	Median	Lower CI	Upper CI	SSef	psrf
Sensitivity of OLBI	0.728	0.594	0.884	25407	1
Sensitivity of the BHAI	0.910	0.778	1.000	26323	1
Specificity of OLBI	0.725	0.550	0.937	26759	1
Specificity of the BHAI	0.836	0.602	1.000	22614	1
Prevalence (Belgium)	0.519	0.302	0.714	21574	1
Prevalence (Switzerland)	0.818	0.592	1.000	23343	1
Covariance sensitivity	0	0	0	NA	NA
Covariance specificity	0	0	0	NA	NA

Table S8 model without conditional dependency and with minimally informative priors but the prior for Specificity of the BHAI (2,1)

	Median	Lower CI	Upper CI	SSef	psrf
Sensitivity of OLBI	0.728	0.594	0.884	25407	1
Sensitivity of the BHAI	0.910	0.778	1.000	26323	1
Specificity of OLBI	0.725	0.550	0.937	26759	1
Specificity of the BHAI	0.836	0.602	1.000	22614	1
Prevalence (Belgium)	0.519	0.302	0.714	21574	1
Prevalence (Switzerland)	0.818	0.592	1.000	23343	1
Covariance sensitivity	0	0	0	NA	NA
Covariance specificity	0	0	0	NA	NA

Table S9 model without conditional dependency and with informative priors for OLBI when compared to MBI

	Median	Lower CI	Upper CI	SSef	psrf
Sensitivity of OLBI	0.715	0.597	0.837	27350	1
Sensitivity of the BHAI	0.817	0.710	0.928	29441	1
Specificity of OLBI	0.807	0.635	1.000	28652	1
Specificity of the BHAI	0.854	0.629	1.000	26202	1
Prevalence (Belgium)	0.598	0.398	0.773	26277	1
Prevalence (Switzerland)	0.877	0.687	1.000	26336	1
Covariance sensitivity	0	0	0	NA	NA
Covariance specificity	0	0	0	NA	NA

Table S10 model without conditional dependency and with informative priors for OLBI when compared to lDPESQ-14

	Median	Lower CI	Upper CI	SSef	psrf
Sensitivity of OLBI	0.696	0.539	0.876	17814	1
Sensitivity of the BHAI	0.881	0.700	1.000	11708	1
Specificity of OLBI	0.672	0.463	0.889	26088	1
Specificity of the BHAI	0.786	0.501	1.000	17786	1
Prevalence (Belgium)	0.519	0.276	0.760	18540	1
Prevalence (Switzerland)	0.821	0.541	1.000	18846	1
Covariance sensitivity	0.028	-0.020	0.118	13724	NA
Covariance specificity	0	0	0	NA	NA

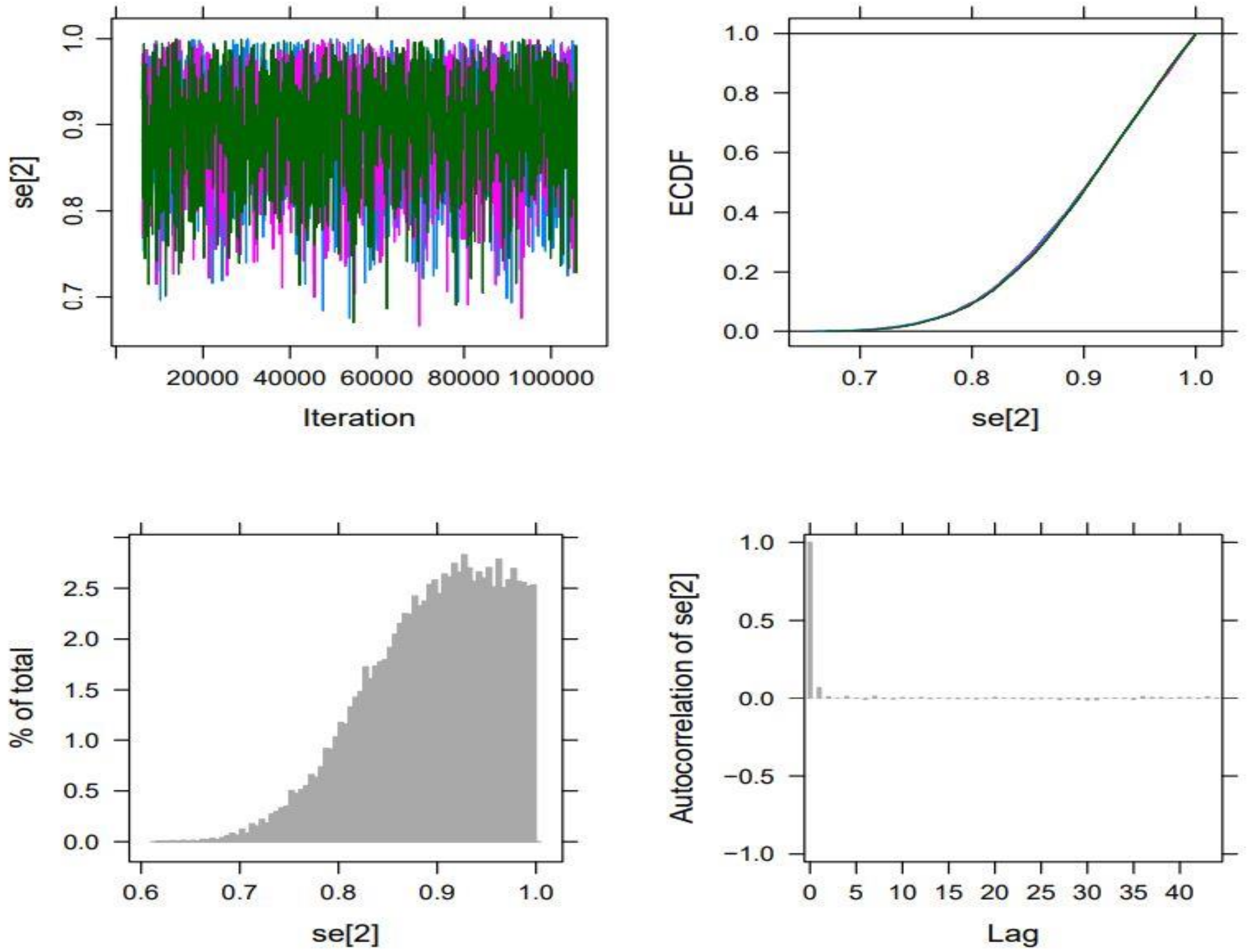

Figure S1. The results of the Bayesian latent class modelling with minimally informative priors and without conditional dependency to estimate the sensitivity of BHAI

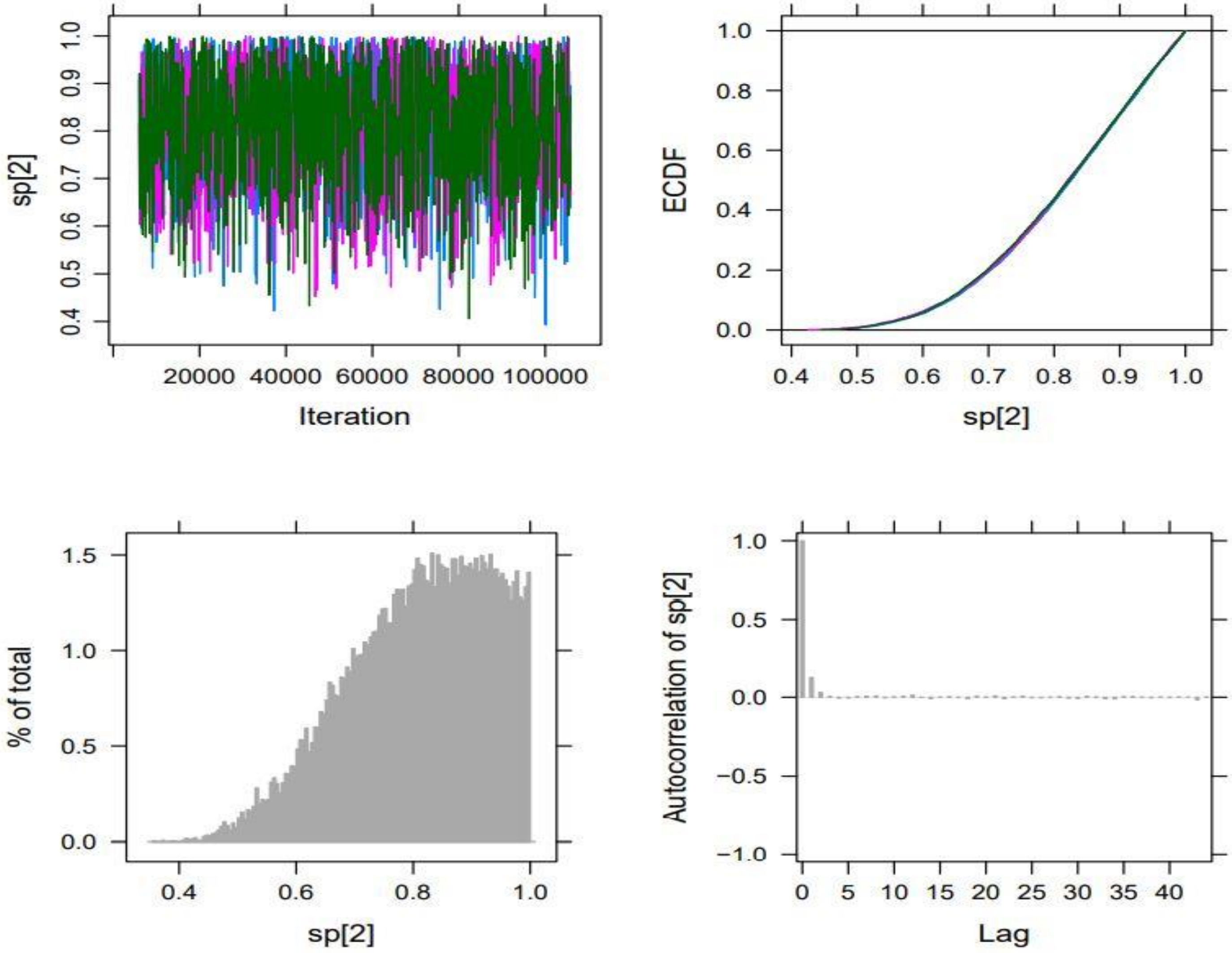

Figure S2. The results of the Bayesian latent class modelling with minimally informative priors and without conditional dependency to estimate the specificity of BHAI
